# Pulse Transit Time Based Continuous Cuffless Blood Pressure Estimation: A New Extension and A Comprehensive Evaluation

**DOI:** 10.1038/s41598-017-11507-3

**Published:** 2017-09-14

**Authors:** Xiaorong Ding, Bryan P. Yan, Yuan-Ting Zhang, Jing Liu, Ni Zhao, Hon Ki Tsang

**Affiliations:** 1 Department of Electronic Engineering, The Chinese University of Hong Kong, Hong Kong, China; 2 Department of Medicine and Therapeutics, The Chinese University of Hong Kong, Hong Kong, China; 3 Yuan-Ting Zhang is with Key Laboratory for Health Informatics of the Chinese Academy of Sciences (HICAS), Shenzhen Institutes of advanced technology, Shenzhen, China

## Abstract

Cuffless technique enables continuous blood pressure (BP) measurement in an unobtrusive manner, and thus has the potential to revolutionize the conventional cuff-based approaches. This study extends the pulse transit time (PTT) based cuffless BP measurement method by introducing a new indicator – the photoplethysmogram (PPG) intensity ratio (PIR). The performance of the models with PTT and PIR was comprehensively evaluated in comparison with six models that are based on sole PTT. The validation conducted on 33 subjects with and without hypertension, at rest and under various maneuvers with induced BP changes, and over an extended calibration interval, respectively. The results showed that, comparing to the PTT models, the proposed methods achieved better accuracy on each subject group at rest state and over 24 hours calibration interval. Although the BP estimation errors under dynamic maneuvers and over extended calibration interval were significantly increased for all methods, the proposed methods still outperformed the compared methods in the latter situation. These findings suggest that additional BP-related indicator other than PTT has added value for improving the accuracy of cuffless BP measurement. This study also offers insights into future research in cuffless BP measurement for tracking dynamic BP changes and over extended periods of time.

## Introduction

Cardiovascular disease (CVD) is the leading cause of death globally, with stroke being the second biggest contributor. Both types of diseases are associated with poor prognosis, increased mortality and heavy burden on the health care. Hypertension remains the single most important modifiable risk factor for both CVD and stroke, and it is well recognized that the adverse cardiovascular consequences largely depend on high blood pressure (BP) levels^1^. In addition to absolute BP values, evidence from observational studies and post-hoc analysis of data from clinical trials indicates that these outcomes could also depend on increased BP variability (BPV)^2^. Increased short-term and long-term BPV are associated with the development, progression, and severity of cardiac, vascular, and renal damage and with a high risk of cardiovascular events and mortality. It is therefore crucial to minimize the risk by monitoring the changes of BP and maintaining control in the early stage.

The current most common noninvasive method of measuring BP relies either on the auscultatory method or on the oscillometric approach that requires an inflatable cuff, which may cause discomfort and could only provide intermittent BP readings. Though arterial tonometry and volume clamp technique are available for continuous BP monitoring, they are bulky and partly intrusive, which have hampered their widespread application. Noninvasive 24-hour ambulatory BP monitoring allows estimates of BP-related factors for cardiac events. The factors include, for example, the abnormal BP variability or profiles of circadian variability, which can be obtained with ambulatory BP being usually measured at regular intervals, e.g., every 30 min. Nevertheless, such ambulatory device cannot avoid using the cuff, and to some extent has been too bulky for wearables. There is therefore a very high demand for the development of novel technologies to monitor BP continuously and unobtrusively without a cuff. For decades, researchers have attempted to develop cuffless technique for BP measurement. Methods are mainly based on pulse wave velocity (PWV)/pulse transit time (PTT) and pulse wave analysis (PWA), where the pulse, e.g. electrocardiogram (ECG), photoplethysmogram (PPG) and ballistocardiogram (BCG), originates from the cardiovascular system and can be obtained noninvasively and even unobtrusively.

The technology of cuffless BP has been attractive in recent years, and there are a wide variety of studies in terms of sensing^3, 4^, signal processing^5^, calibration or modeling^6^, and performance validation^7, 8^. Among the different methods being developed for cuff-free BP measurement, PTT method has shown great potential and attracted much attention over the years^6^. There are a few commercially available devices that are based on this method, including the FDA cleared Sotera ViSi Mobile continuous noninvasive BP (cNIBP) monitoring and the SOMNOtouch NIBP systems^9^. Although it is promising, there are still many challenges ahead for the extensive clinical application^6, 10^. The accuracy is one of the key issues to address. Previous studies have proposed and developed various calibration models to translate the PTT or BP-related indicators to BP. These approaches mainly include application of the Moens-Korteweg (M-K) formula, heuristic modeling with regression technique, or predictive modeling with data-driven methods such as machine learning. For example, early studies by Chen *et al*.^11^ and Poon *et al*.^12^ have developed PTT-BP models based on the M-K equation, and their results demonstrated that PTT is able to track BP with quite promising accuracy. Most of the studies on heuristic modeling used linear or nonlinear regression, to adapt indirect indicators to BP, where the indicators include PTT or extra parameters, such as heart rate (HR)^13^. The modeling can be derived by simple regression technique or advanced machine learning method. Muehlsteff *et al*.^14^ investigated various calibration models to estimate BP, such as linear logarithmic function and inverse (square) function, and found that inverse square function achieved the lowest error with intra root mean square error of 3.6 mmHg. Further to explore more comprehensive indicators that are available in ECG or PPG signals, researchers have analyzed pulse wave morphology and employed machine learning method to obtain model for better describing the relationship between BP-indicators and BP^15–17^. One study by Monte-Moreno *et al*. estimated BP with sole PPG waveform with random forest technique, and achieved the correlation coefficients between reference and prediction value of 0.91 and 0.89 for systolic BP (SBP) and diastolic BP (DBP), respectively^15^. However, most of these studies focus on using only PTT to indicate BP, and the translation from PTT to BP has been implemented through regression method. Moreover, the majority of studies were validated only with subject at rest state within a short period of time, e.g., minutes to hours. There is a need for a more comprehensive study to evaluate the performances of various models, the performances in monitoring dynamic BP variations induced by exercise, and the performances over longer calibration intervals.

In this study, we assess the accuracy of a new model with an extra indicator – PPG intensity ratio (PIR), which has been demonstrated to reflect the arterial vasomotion and thereby slow variations of BP in our previous studies^18, 19^. The model combines PIR and PTT to estimate beat-by-beat BP from the physiological perspective. We experimentally compare our methods that used PTT and PIR, with other six commonly used PTT models. We then analyze the BP estimations of those methods for different subject group, while at rest and performing different maneuvers, and over an extended period of time.

## Results

The performances of cuffless BP estimations using the methods with PTT and PIR and methods with sole PTT are firstly compared for all subjects (19 normotensive and 14 hypertensive) at rest. Then, the performances of these methods are evaluated for normotensive and hypertensive subjects, respectively. The comparisons are also analyzed with BP changes elicited by various maneuvers, i.e., active standing (AS), deep breathing (DB), Valsalva maneuver (VM), and sustained handgrip (HG). Finally, their performance with a calibration interval of 24 hours will be presented.

### Overall Comparison

The overall performance of the proposed methods as well as the compared methods were validated over 5024 beats from 33 subjects (19 normotensive and 14 hypertensive) with subject supine and seated at rest. The values of SBP and DBP during the monitoring period for all the samples were 132.99 ± 17.94 mmHg and 75.12 ± 12.04 mmHg, respectively.

**Figure 1:**
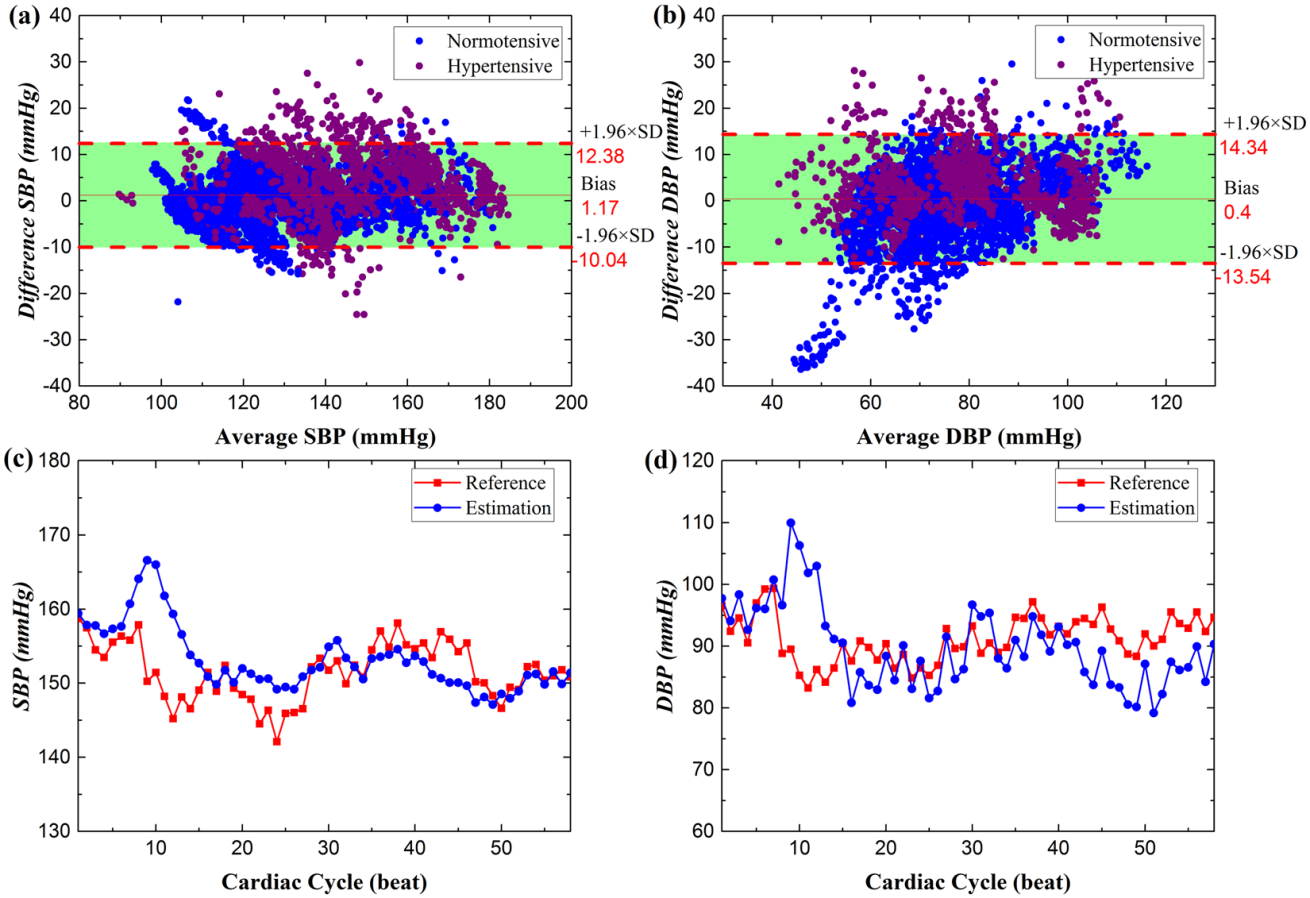
The Bland-Altman plots of the overall BP estimation with the proposed method against the reference method for (**a**) SBP and (**b**) DBP. And one representative (**c**) SBP and (**d**) DBP estimation (blue) versus reference (red) of one hypertensive subject seated at rest state.

The performance of the proposed method PTT&PIR#2 for SBP and DBP estimations against the reference BP that were measured with Finometer are shown in Fig. 1. The estimation errors for SBP and DBP were 1.17 ± 5.72 mmHg and 0.40 ± 7.11 mmHg, respectively. We may observe from the Bland-Altman plots that the estimations agreed well with the reference, with 95% of the differences lie within the agreement area. Further, the differences of BP estimation for the normotensive group agreed better with the reference BP than with that of the hypertensive group. One specific sample, as shown in Fig. 1c and d, further demonstrates that the estimation is able to track BP, with the estimation error of 1.62 ± 4.84 (3.52) mmHg and −1.29 ± 7.22 (5.62) mmHg for SBP and DBP, respectively. For this proposed method, the SBP estimation was more accurate than DBP estimation.

**Figure 2:**
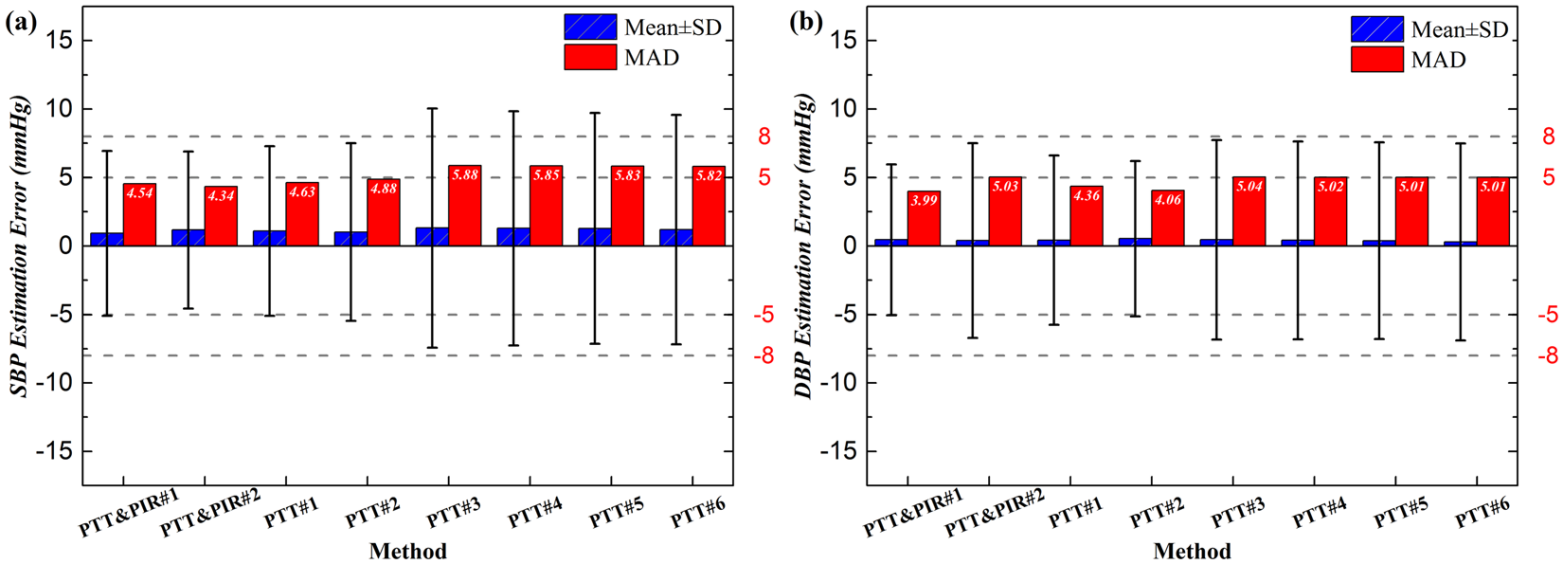
The overall comparison of different methods for (**a**) SBP and (**b**) DBP measurement.

The estimation errors of the proposed methods with PTT and PIR were compared with those with sole PTT for SBP and DBP, and the results are illustrated in Fig. 2. For SBP (Fig. 2a), the mean of the estimation errors by all methods were comparable, F(7, 40184) = 1.72, *p* = 0.098, being around 1 mmHg and within 5 mmHg, while their SD of the errors were significant, F(7, 40184) = 89.41, *p* = 0.000. Among the proposed methods and the compared methods, PTT&PIR#2 worked the best, with its estimation precision about 0.3 mmHg higher than that of PTT&PIR#1, and around 0.5–3 mmHg higher than all the other comparison methods. The methods using PTT and PIR, as well as PTT#1 and PTT#2, performed better than PTT#3-PTT#6, with the SD of the error bias within 8 mmHg and MAD within 5 mmHg. It is noted that there was no significant difference among the estimations within methods PTT#3-PTT#6, and the SD of the estimation biases exceeded 8 mmHg.

Similarly, for DBP as shown in Fig. 2b, the means of the estimation error by all the methods were not significantly different, F(7, 40184) = 0.60, *p* = 0.757, whereas the SD of the estimation errors were significantly different at the 0.05 level, F(7, 40184) = 50.84, *p* = 0.000. ﻿However, the values of the error means and SD were within 5 mmHg and 8 mmHg for all those methods. The MAD of the estimation errors were also significantly different among these methods, F(7, 40184) = 48.37, *p* = 0.000, among which PTT&PIR#1 had a significant lowest MAD than all other methods. Likewise, there were no significant differences among those regression methods, i.e., PTT#3-PTT#6, and they were not as good as the methods with PTT and PIR as well as PTT#1 and PTT#2.

### Comparison between Normotensive and Hypertensive Group

**Figure 3:**
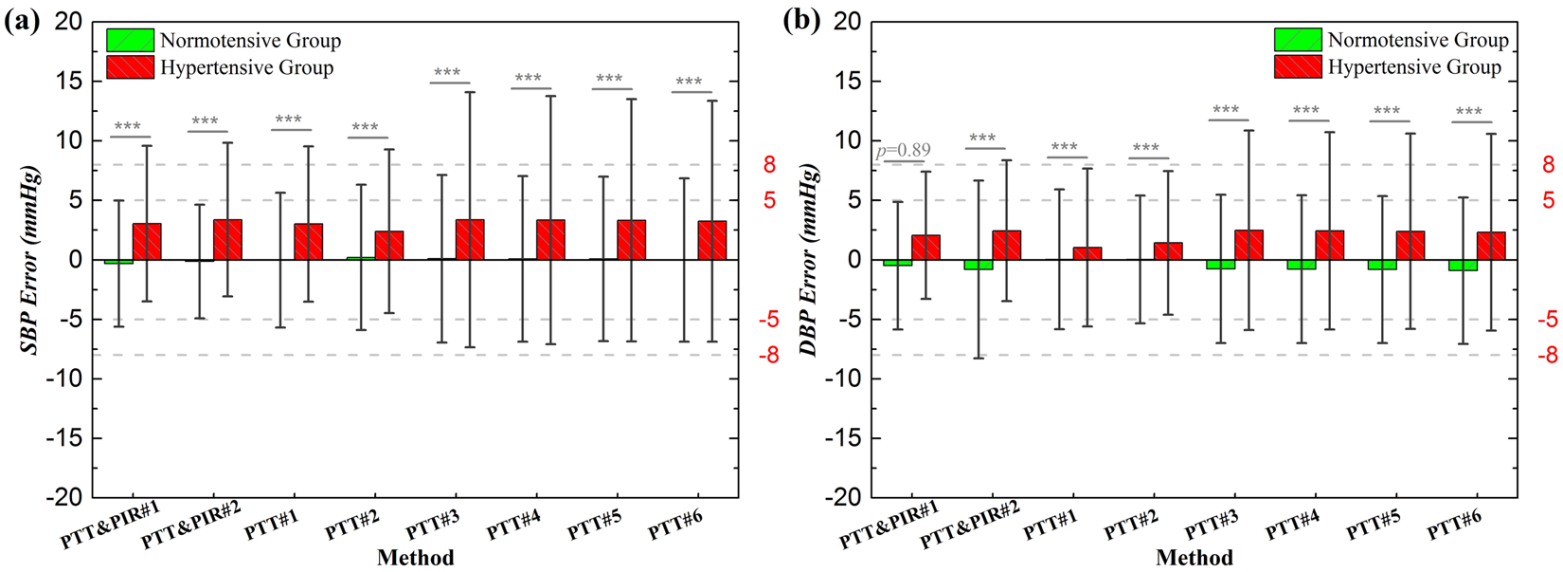
Performance comparison in normotensive group and hypertensive group for (**a**) SBP and (**b**) DBP measurement.

The performance of these methods was evaluated separately for the normotensive group and the hypertensive group, and the results are illustrated in Fig. 3. There were 3160 beats of estimation for normotensive group, with the reference average SBP and DBP of 128.75 ± 16.39 mmHg and 68.73 ± 11.09 mmHg, respectively. And there are 1864 beats of estimations for the hypertensive group, with the reference average SBP and DBP of 136.67 ± 17.40 mmHg and 74.80 ± 12.82 mmHg, respectively. The estimation errors of the hypertensive group were significantly higher than those of the normotensive group, for both SBP and DBP, and for all the methods, except for PTT&PIR#1 when estimating DBP. The error bias of hypertensive group was larger than that of the normotensive group for all the methods, so was the SD of the bias, indicating that the estimations for hypertensive subjects might be overestimated and the variations in individuals were higher. The discrepancies were particularly obvious in SBP estimation.

### Comparison of Different Maneuvers

**Figure 4:**
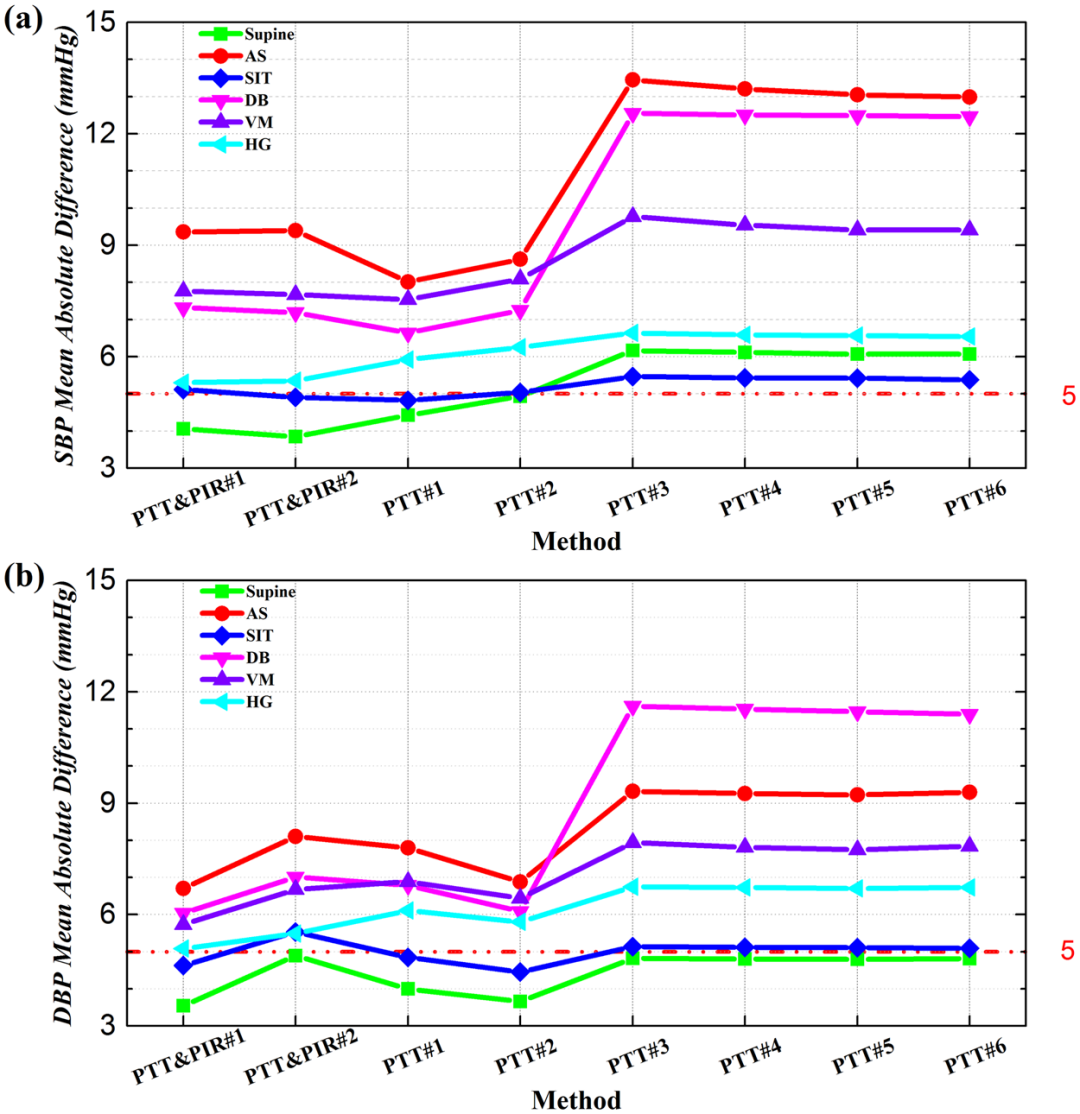
Performance comparison for different maneuvers for (**a**) SBP and (**b**) DBP measurement.

The performance of these methods with subjects at different positions and undergoing different maneuvers was analyzed. The BP estimation errors evaluated by MAD are presented in Fig. 4. We can see that the performance of those methods varied at different statuses. While supine at rest, PTT&PIR#2 was the most accurate for SBP estimation, meanwhile PTT&PIR#1 the best for DBP estimations. While sitting at rest, PTT#1 worked the best for estimating SBP, but there was no significant difference between PTT#1 and PTT&PIR#2. For DBP estimation, PTT#2 as well as PTT&PIR#1 were superior to the others. For AS, DB, and VM, when there were dynamic BP changes, PTT&PIR#1 and PTT&PIR#2 performed comparably with PTT#1 and PTT#2 (*p* = 0.17, *p* = 0.85, *p* = 0.15, *p* = 0.83), and they significantly outperformed the four regression methods, though the estimation errors were beyond 5 mmHg for all those methods. And for HG, the performance of PTT&PIR#1, PTT&PIR#2, and PTT#1 were comparable, and superior to the remaining methods.

**Figure 5:**
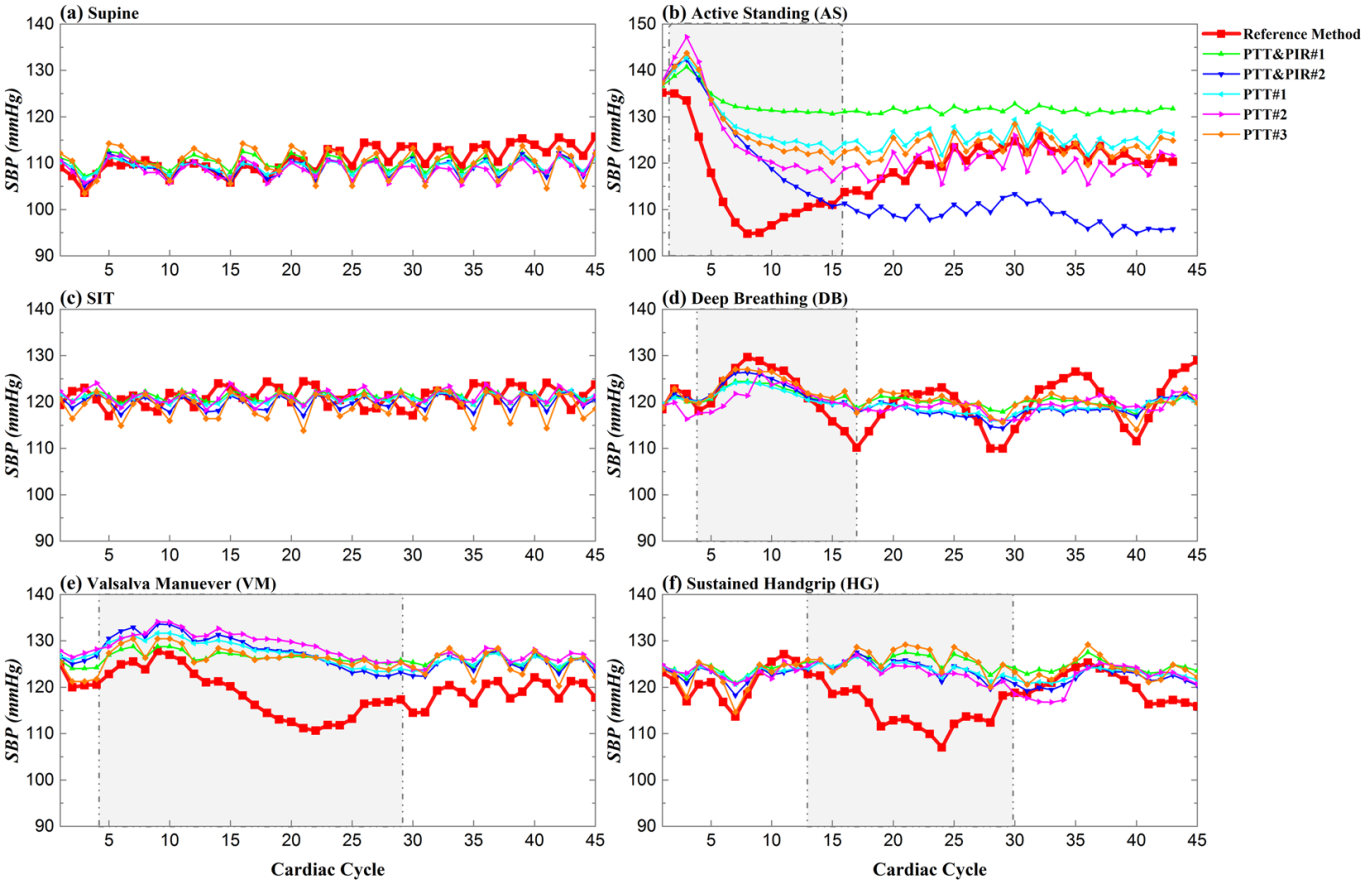
A representative beat-to-beat SBP estimation by different methods against reference SBP measured by Finapres at different maneuvers: (**a**) supine, (**b**) active standing, (**c**) sit, (**d**) Valsalva maneuver, (**e**) deep breathing, and (**f**) sustained handgrip.

Figure 5 shows one representative beat-to-beat SBP estimations for these methods under different states. The cuffless methods tracked SBP well at rest state either with supine or seated positions. However, large deviations existed between the estimations and the reference when BP changes were elicited by AS (Fig. 5b), DB (Fig. 5d), VM (Fig. 5e) and HG (Fig. 5f).

### Comparison of Different Calibration Intervals

**Figure 6:**
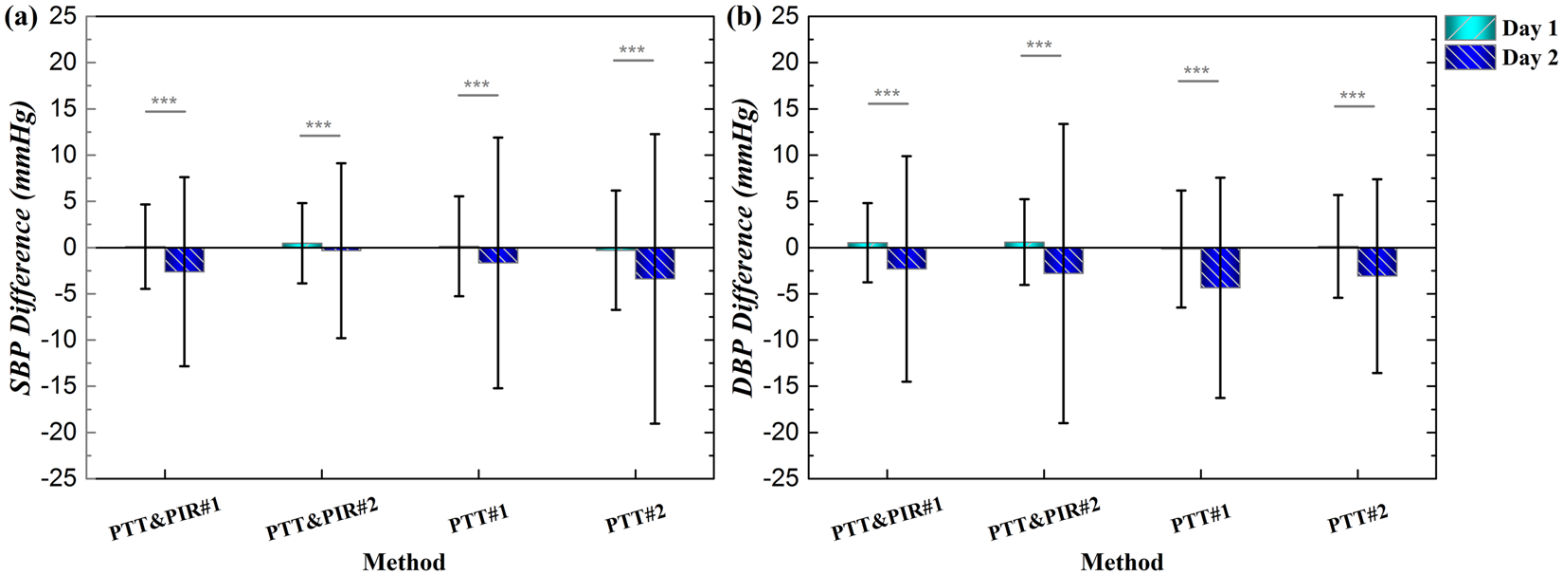
Performance comparison at different days for (**a**) SBP and (**b**) DBP measurement with the proposed methods, i.e., PTT&PIR#1 and PTT&PIR#2, and the compared methods, PTT#1 and PTT#2.

To validate and compare the performance of the proposed and compared methods over an extended calibration interval, e.g., 24 hours, the initial calibrations of these models were conducted at the beginning of the first day measurement. The errors of SBP and DBP estimations using the methods with PTT and PIR as well as the two best of the methods that used sole PTT, on the first day (Day 1) and second day (Day 2) after the calibration are shown in Fig. 6. Noticeably, the estimation errors of SBP and DBP by either the methods with PTT and PIR or the methods with sole PTT were significantly increased at Day 2 after the initial calibration, with both the bias and the SD of the bias increased. For SBP, the estimation accuracy of PTT&PIR#2 remained better in the Day 2, with the SD of the discrepancy increased by around 5 mmHg. PTT&PIR#1 was as good as PTT&PIR#2. However, the SD of the estimation bias for PTT#1 and PTT#2 approximately raised, from 5 mmHg to 13 mmHg. For DBP, estimations by PTT#1 performed better than all the others the current day of the calibration, however the SD surged from 4 mmHg to 12 mmHg at Day 2. And PTT#2 worked the best at Day 2, with the increment of the SD of the estimation error at around 5 mmHg.

For SBP in Day 2, the means of the estimation errors were comparable, except that the error mean of PTT#2 was significantly higher than the PTT&PIR#2. In addition, the SD of the estimation errors were significantly different, F(3, 1336) = 39.53, *p* = 0.000. For DBP in Day 1 and Day 2, there is no significant differences between the estimation biases, but their SD was significantly different, Day 1: F (3, 1256) = 19.76, *p* < 0.001; Day 2: F (3, 1336) = 28.03, *p* = 0.000.

## Discussion

PTT method has been the most commonly employed technique for cuffless BP measurement. However, previous studies have demonstrated that sole PTT is unreliable to offer full insights into BP variations in different situations. The introduction of extra parameter that is indicative of BP changes is expected to improve the methods using single PTT. We used PIR and PTT to establish models for BP estimation, and compared them with approaches using single PTT. Experimental results show that the proposed methods overall achieved better estimation accuracy than methods with sole PTT with subjects at rest. Furthermore, they were more accurate for measurements on either normotensive or hypertensive group, for estimations under dynamic cardiovascular changes, and for long-term monitoring over 24 hours.

For the overall performance, the proposed methods with both PTT and PIR had the lowest errors of 1.17 ± 5.72 (4.34) mmHg and 0.46 ± 5.49 (3.99) mmHg for estimation of SBP and DBP, respectively. This indicates that it would beneficial to introduce extra BP indicators for improving the estimation accuracy. Among the methods that use sole PTT, PTT#1 and PTT#2 were superior to PTT#3-PTT#6, revealing that the models that were derived based on M-K equation were more efficient than those based on regression methods. Further, methods PTT#3-PTT#6 provided very similar estimation accuracy with SD of the errors beyond 8 mmHg for SBP. This demonstrate that it is inadequate to map PTT to BP with regression technique. One major limitation of the regression model is that it cannot adequately reflect the variability of BP if the regression function does not involve the variable that is indicative of BP changes. This is the reason why the estimation accuracy would be improved when extra parameter except for PTT are introduced, e.g., HR. For example, Kim *et al*. has added HR and another indicator of arterial stiffness that was calculated from PPG as the duration from the maximum derivative point to the dicrotic peak of PPG signal. With the combination of these indicators and PTT via multiple regression analysis, the performance of BP estimation was significantly enhanced^20^. The regression method also bears other shortcomings. For instance, it is too simple to reflect the complicated regulation of the cardiovascular system.

The proposed methods worked better than the compared methods on either normotensive or hypertensive group. The estimation errors were significantly higher in hypertensive subjects for all those methods. This is in agreement with our earlier study^21^, which showed that the overall accuracy of SBP estimation was significantly lower in subjects with hypertension and heart diseases than in healthy subjects. However, except for the evidence that the SD of the estimation errors of PTT#3-PTT#6 went beyond 8 mmHg in hypertensive group, all the other estimations error biases and SD were within 5 ± 8 mmHg. These reveal the potential of those PTT methods for monitoring continuous BP for normotensive users. It is also noteworthy that DBP estimation achieved comparable accuracy on those two groups using the method PTT&PIR#1, indicating this method is applicable for either hypertensive or normotensive individuals. For hypertensive group, the mean error bias for both SBP and DBP estimations were negative, indicating the BP of this group was overestimated. Since the arterial stiffness is higher on average in the hypertensive group than in the normotensive group, one possible reason for the overestimation is the improper mapping from PTT to BP in this group. This suggests that it may be valuable to explore the calibration model on diverse groups to obtain a more precise estimate of a wide range of population.

Performance validation with dynamic BP change induced by various cardiac maneuvers (i.e., AS, DB, VM and HG) showed that the cuffless estimations of BP were deviated more from the reference BP compared to that at rest condition, with the MAD exceeded 5 mmHg for all methods. For SBP estimation, PTT#1 and PTT#2 were on the whole better than other methods, with the estimation difference at those dynamic situations were less, and this occurred in PTT&PIR#1 for DBP estimation. These findings reveal the inadequacy of those methods to follow the dynamic changes in BP elicited by the cardiovascular autonomic nervous activities. When there is stimulus to the cardiovascular system, there is a series of reactions which causes complex response that ultimately changes the cardiac output, peripheral resistance and BP. As can be observed from Fig. 5a and c, while at rest with different positions, BP fluctuated mainly due to the spontaneous respiration, and the estimations could track well the fluctuations. By contrast during cardiac maneuvers, other than the respiratory-induced oscillations, there were dynamic changes of the slow variations in BP. During AS (Fig. 5b), a transit but large drop of SBP was caused at the initial phase due to a reduction total peripheral resistance. This change can be explained by the integrated effects of changes in intro-abdominal pressure and hence volume of venous return, cardiopulmonary baroreflex sensitivity and other possible influences^22^. Cuffless estimations can, to some extent, track the drop but fail to recover, indicating the parameters – PTT and PIR, or the established models is unable to reflect the changes in the overall peripheral resistance or stroke volume. For DB (Fig. 5d), these methods were able to trace the trend during inhalation and exhalation, but the changes in level cannot be followed. As the pressure change during DB involves cardiopulmonary activity that cannot be well reflected by the BP indicators in this study, the estimations thus cannot be accurately obtained. And the responses of BP to VM were characterized by a pronounced reduction of BP, which elicited the sympathetic-mediated vasoconstrictor response resulting in BP recovery via baroreflex^23^. Compared with other maneuvers, estimations of BP changes in HG were more accurate. Usually during HG, there is a small decrease in BP that is possibly due to a reduction in peripheral vascular resistance as well as a decline in cardiac output. Since PIR is potential to evaluate the peripheral resistance, the DBP estimation with PIR was significantly better than other methods. Usually, the transit changes in BP are related the baroreflex regulation, and HR is an efficient variable to evaluate this activity. Thus, it would be better to involve HR in this kind of dynamic situation.

The extension of the calibration interval to 24 hours indicate that the estimation accuracy substantially decreased the second day after the initial calibration, for all those methods. However, the proposed methods still performed better for SBP estimation than those of the other methods at the second day. The proposed methods, particularly PTT&PIR#1, were not as good as PTT#2 for DBP estimation on Day 2. This can be explained by the fact that the variability of DBP is low while PIR and PTT track mainly the LF and HF variations of BP respectively, and the combination of PIR and PTT for DBP was not able to work well for a long period of time.

There are several limitations of this investigation. First, the reference BP was measured with Finapres system. Though it is reported to be able to track dynamic change, its accuracy for measuring absolute BP amplitude remains controversial. There has been a study using advance volume-compensation method to improve the accuracy of such method, which may be used in further study to reduce the error for reference measurement^24^. Second, performance of extended calibration interval was only validated on a small sample size, and it has not been conducted in the hypertensive groups. Although there is much to be done, our work generates important findings in the field of cuffless BP measurement. To move forward the goal to measure continuous BP with acceptable accuracy over a longer period of time and at various dynamic conditions, future efforts should, on the one hand, focus upon the exploration of new features that can characterize the BP changes. On the other hand, dynamic adaptive model rather than static model should be considered to reflect the regulation mechanism of BP.

## Conclusion

In this work, we have investigated the accuracy of PIR with PTT for cuffless continuous BP monitoring. The methods using PIR and PTT were compared with other commonly used PTT algorithms in normotensive and hypotensive subjects, with subjects at different positions, undergoing various maneuvers, and over an extended period beyond 24 hours after calibration. In general, the PIR with PTT approach outperformed the other comparison methods, though the ability of tracking abrupt changes in BP that are regulated by the autonomic nervous activities should be further addressed. The accuracy of extended calibration interval remains to be resolved. The potential of PIR with PTT technique for unobtrusive monitoring of BP for ambulatory or home healthcare is significant. Although cuffless technique holds great promise, the true potential of this approach will be realized only when efficacy and effectiveness studies show that its application can reduce the incidence of hypertension and improve the cardiovascular health of populations.

## Methods

### PTT Measurement and Preprocessing

We calculated PTT as the time difference between the R wave peak of the ECG signal and the peak of the first derivative of the PPG signal (dPPG). High and low frequency noise in ECG and PPG signals were removed with zero-phase filter and wavelet denoising method. For the latter method, we used wavelet Daubechies 3 (db3) to decompose the signal at level 16 and then reconstructed without the noise component.

### PIR Derivation and Cuffless BP models

**Figure 7:**
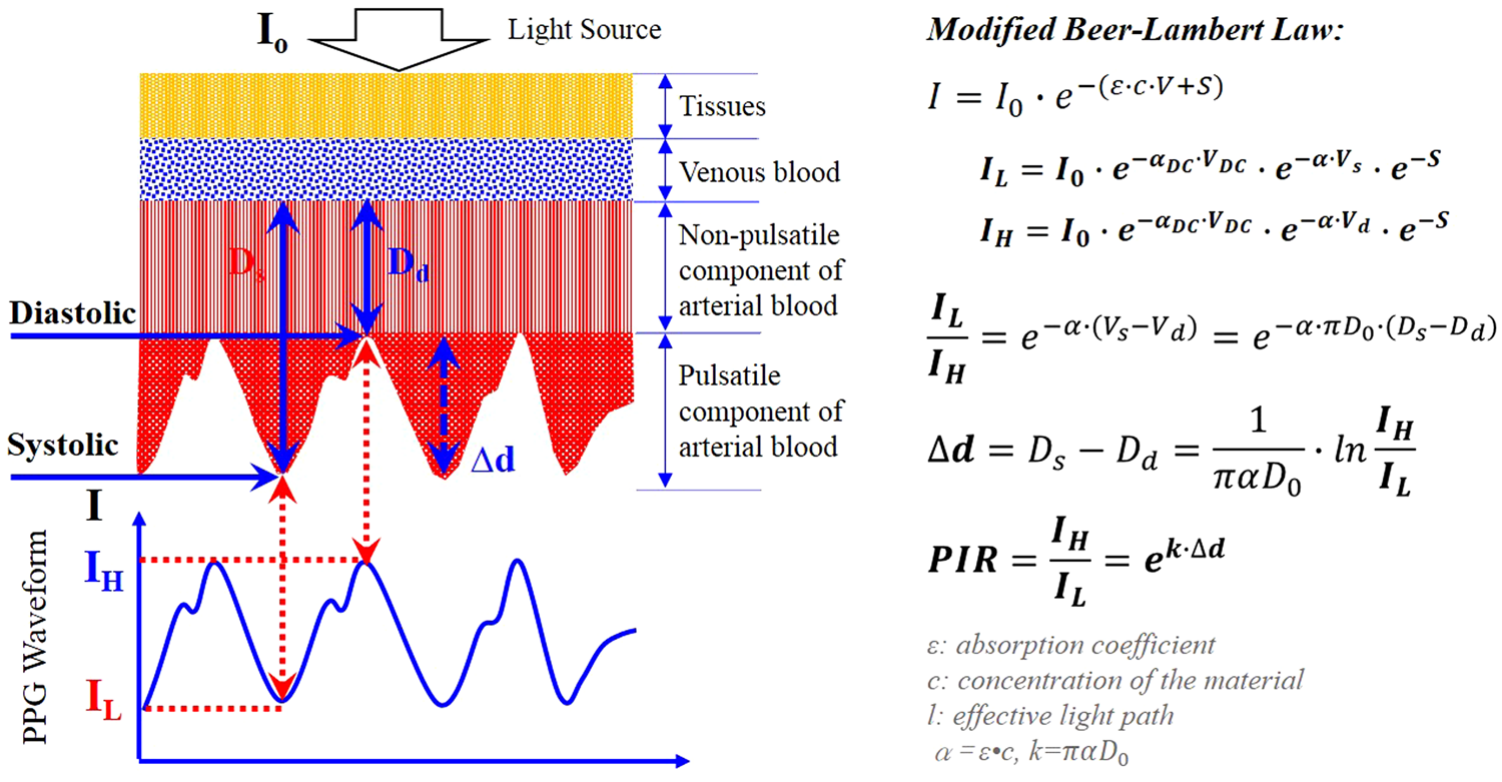
Diagram for derivation of PPG intensity ratio (PIR).

With PPG signal collected from a peripheral artery as shown in Fig. 7, the incident light intensity *I*_0_ of the light emitter will become *I* detected by the light detector after the transmission through the tissue, venous blood, and arterial blood. It should be noted that the arterial blood contains non-pulsatile component as well as pulsatile component. During diastole, the arterial diameter is at its minimal level *D*_*d*_, and consequently the transmitted light intensity will be at the highest level *I*_*H*_. On the contrary, the transmitted light intensity will decrease to the minimal level *I*_*L*_ during systole as a result of the increase of arterial diameter from the minimal *D*_*d*_ to maximal *D*_*s*_. During one cardiac cycle, the change of the optical path is corresponding to the diameter change *Δd*. Therefore, the diameter change can be derived from the peak and valley intensity of the PPG signal. The detected PPG signal consists of DC component mainly due to the light absorption of the tissue, the venous blood and non-pulsatile blood, while the alternating component is generated because of the blood pulsation. According to the modified Beer-Lambert law^25, 26^, PIR – ratio of PPG peak intensity *I*_*H*_ to valley intensity *I*_*L*_ can be derived in terms of the arterial diameter change under the assumption that the characteristic parameter α keeps constant and there is seldom change of the baseline arterial diameter, as explained in Fig. 7.

Our preliminary study indicates that MBP can be correlated with PIR, as PIR can potentially reflect the peripheral resistance^27, 28^. Further based on the Bramwell and Hill (B-H) equation^29^:

1 $${\rm{PWV}}=\sqrt{\frac{{\rm{V}}}{{\rm{\rho }}\mathrm{dV}/{\rm{dP}}}}$$

which relates PWV to arterial compliance dV/dP, blood density ρ and volume V, and the consideration that the arterial pressure change in one cardiac cycle is pulse pressure (PP), and the volume change is approximately the sectional area change ∆A which can be further expressed in terms of arterial diameter change ∆d, (2) can be obtained accordingly.

2 $$\frac{{\rm{\Delta }}{\rm{V}}}{{\rm{V}}\cdot {\rm{\Delta }}{\rm{P}}}=\frac{{\rm{\Delta }}{\rm{A}}}{{\rm{A}}\cdot {\rm{PP}}}=\frac{{{{\rm{D}}}_{{\rm{s}}}}^{2}-{{{\rm{D}}}_{{\rm{d}}}}^{2}}{{{{\rm{D}}}_{{\rm{d}}}}^{2}\cdot {\rm{PP}}}=\frac{{{\rm{D}}}_{{\rm{s}}}+{{\rm{D}}}_{{\rm{d}}}}{{{{\rm{D}}}_{{\rm{d}}}}^{2}}\cdot \frac{{\rm{\Delta }}{\rm{d}}}{{\rm{PP}}}$$

Substituting PWV = L/PTT and (2) into (1), we have:

3 $$\frac{{\rm{L}}}{{\rm{PTT}}}=\sqrt{\frac{1}{{\rm{\rho }}\cdot ({{\rm{D}}}_{{\rm{s}}}+{{\rm{D}}}_{{\rm{d}}})/{{{\rm{D}}}_{{\rm{d}}}}^{2}}\cdot \frac{{\rm{PP}}}{{\rm{\Delta }}{\rm{d}}}}$$

As a result, PP can be derived in terms of ∆d and PTT:

4 $${\rm{PP}}=\frac{{{\rm{\rho }}{\rm{L}}}^{2}({{\rm{D}}}_{{\rm{s}}}+{{\rm{D}}}_{{\rm{d}}})}{{{{\rm{D}}}_{{\rm{d}}}}^{2}}\cdot {\rm{\Delta }}{\rm{d}}\cdot \frac{1}{{{\rm{PTT}}}^{2}}$$

Under the assumption that the parameters such as ρ, L, D_s_, D_d_, and α keep constant, ∆d is approximately linear with PIR, i.e., ∆d ∝ PIR. Therefore, (5) can be obtained.

5 $${\rm{PP}}\propto \frac{{\rm{PIR}}}{{{\rm{PTT}}}^{2}}$$

And ultimately PP can be derived with the calibrated PP, PIR and PTT, i.e., PP0, PIR_0_, and PTT_0_.

6 $${\rm{PP}}={{\rm{PP}}}_{0}\cdot \frac{{\rm{PIR}}}{{{\rm{PIR}}}_{0}}\cdot {(\frac{{{\rm{PTT}}}_{0}}{{\rm{PTT}}})}^{2}$$

Finally, SBP and DBP can be derived with PIR and PTT accordingly.

7 $$SB{P}_{i}=MB{P}_{0}\cdot \frac{PI{R}_{0}}{PI{R}_{i}}+\frac{2}{3}\cdot \frac{PI{R}_{i}}{PI{R}_{0}}\cdot {(\frac{PT{T}_{0}}{PT{T}_{i}})}^{2}$$

8 $$DB{P}_{i}=MB{P}_{0}\cdot \frac{PI{R}_{0}}{PI{R}_{i}}-\frac{1}{3}\cdot \frac{PI{R}_{i}}{PI{R}_{0}}\cdot {(\frac{PT{T}_{0}}{PT{T}_{i}})}^{2}$$

where the subscript of *i* and zero indicate the *i*th heart beat and the initial calibration value, respectively.

The proposed method is compared with our previously established PIR model – PTT&PIR#1^19^, and other six commonly used PTT methods, including the two most cited previous works that are based on PTT and four commonly studied regression methods. They are listed in Table 1.

**Table 1:** Comparison methods.

	**PTT-BP Model**	
	**SBP**	**DBP**
PTT&PIR#1^19^	${\rm{DBP}}+{{\rm{PP}}}_{0}\cdot {(\frac{{{\rm{PTT}}}_{0}}{{\rm{PTT}}})}^{2}$	${{\rm{DBP}}}_{0}\cdot \frac{{{\rm{PIR}}}_{0}}{{\rm{PIR}}}$
PTT&PIR#2	Eq. (7)	Eq. (8)
PTT#1^11^	$SB{P}_{0}-\frac{2}{\gamma PT{T}_{0}}({\rm{PTT}}-PT{T}_{0})$	$DB{P}_{0}-\frac{2}{\gamma PT{T}_{0}}({\rm{PTT}}-PT{T}_{0})$
PTT#2^12^	${\rm{DBP}}+{{\rm{PP}}}_{0}\cdot {(\frac{{{\rm{PTT}}}_{0}}{{\rm{PTT}}})}^{2}$	$MB{P}_{0}+\frac{2}{\gamma }\,\mathrm{ln}\,\frac{PT{T}_{0}}{PTT}-\frac{1}{3}{{\rm{PP}}}_{0}\cdot {(\frac{{{\rm{PTT}}}_{0}}{{\rm{PTT}}})}^{2}$
PTT#3^30^	${{\rm{a}}}_{3}\cdot {\rm{PTT}}+{{\rm{b}}}_{3}$	${{\rm{a}}}_{3}^{\prime} \cdot {\rm{PTT}}+{{\rm{b}}}_{3}^{\prime}$
PTT#4^31^	${{\rm{a}}}_{4}\cdot {\rm{lnPTT}}+{{\rm{b}}}_{4}$	${{\rm{a}}}_{4}^{\prime} \cdot {\rm{lnPTT}}+{{\rm{b}}}_{4}^{\prime}$
PTT#5^32^	$\frac{{{\rm{a}}}_{5}}{{\rm{PTT}}}+{{\rm{b}}}_{5}$	$\frac{{{\rm{a}}}_{5}^{\prime} }{{\rm{PTT}}}+{{\rm{b}}}_{5}^{\prime}$
PTT#6^14^	$\frac{{{\rm{a}}}_{6}}{{{\rm{PTT}}}^{2}}+{{\rm{b}}}_{6}$	$\frac{{{\rm{a}}}_{6}^{\prime} }{{{\rm{PTT}}}^{2}}+{{\rm{b}}}_{6}^{\prime}$

It should be noted that some models estimated only SBP in the original works, e.g., PTT#1, PTT#3, and their corresponding DBP was estimated in this study in a similar way to that for SBP estimation. Those models were calibrated using Finapres BP and the simultaneously obtained parameters at the beginning of the test. For the proposed models with PTT and PIR, PTT#1 and PTT#2, only one beat of BP, PTT and PIR were used. For the regression models PTT#3 – PTT#6, two beats of BP and PTT were used to derive the coefficients. Thereafter, beat-by-beat BP can be obtained with PTT and PIR. The calibration was conducted individually for each maneuver. For the 24 hours measurement, it was only calibrated at the beginning of the first day measurement. The performance was evaluated against the reference BP measured in the same way in the calibration method.

### Subjects and Experiment

Nineteen healthy normotensive subjects and 14 subjects with hypertension were recruited in this study. All the subjects volunteered to participate and gave their informed consent before taking part in this study. The study was approved by the Institutional Review Board of the Joint Chinese University of Hong Kong – New Territories East Cluster Clinical Research Ethics Committee, Hong Kong, China, and conducted according to Declaration of Helsinki ethical principles for medical research on human subjects. Subject aged between 18–80 years old with a specified range of BP among less than 120 to over 160 mmHg was included. Subject with implantable cardiac devices including permanent pacemakers, cardiac-resynchronization therapy or defibrillator, pregnancy and unable to sign informed consent was excluded. The subject characteristics are illustrated in Table 2.

**Table 2:** Subject characteristics.

	**Subjects (n** = **33)**	**Normotensive Group (n** = **19)**	**Hypertensive Group (n** = **14)**
Mean age (range)	43 (21–77)	26 (21–47)	67 (43–77)
Gender (M/F, n)	20/13	10/9	10/4
Height (cm)	165.9 ± 8.9	167.3 ± 8.2	164.1 ± 9.8
Weight (kg)	64.1 ± 14.6	55.6 ± 10.7	75.7 ± 10.7
Hypertension (n)	14	0	14
*Prehypertension*	7	0	7
*Stage I hypertension*	6	0	6
*Stage II hypertension*	1	0	1
SBP (mmHg)	121.12 ± 19.52	107.74 ± 10.04	139.30 ± 13.50
DBP (mmHg)	68.92 ± 8.16	65.21 ± 6.96	74.00 ± 7.00

**Figure 8:**
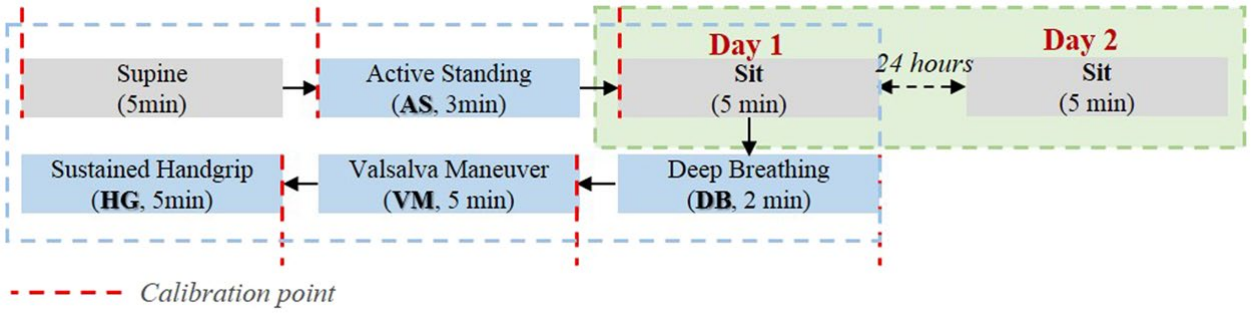
Diagram of the experiment protocol.

The performance of BP estimation was validated with subjects at rest state and with subjects undergoing various maneuvers which are assumed to induce dynamic BP changes. The experimental protocol involves acquiring ECG, PPG, continuous BP simultaneously with subjects at rest while supine, from supine to active standing (AS), as well as during various maneuvers while sitting (Fig. 8). The maneuvers, including deep breathing (DB), Valsalva maneuver (VM), and sustained handgrip (HG) test, have been commonly used in clinical to assess cardiovascular autonomic function^33^. AS was performed 5 min after rest at supine, when the subject was asked to stand up from the supine position and to remain in the standing posture for 3 min. The cyclic DB was performed with the breathing rate of six breaths/min and lasted for two minutes. VM is a sensitive, noninvasive, and widely available clinical test and it provides an indirect index of sympathetic vasoconstrictor functions based on characteristic BP responses. It was performed with moderately forceful attempted exhalation against a closed airway for 20 s, followed by a rest period of one minute. VM was conducted twice continuously; and HG was conducted with continual handgrip at 1/3 of the subject’s maximal strength for 150 s.

ECG was acquired with one-lead ECG electrodes placed on left and right arms, and PPG was recorded with a reflectance type of probe that consists of infrared LED (SFH 4250, 850 nm) and phototransistor (SFH 320) in the left index finger, respectively; and the reference BP was measured by Finometer^®^ (Finapres Medical System, Amsterdam, Netherlands) with the finger cuff on the left thumb and the brachial cuff on the left upper arm. ECG, PPG, and the continuous BP were collected with a sampling rate of 1000 Hz through DATAQ and imported to computer for ad-hoc processing.

To investigate the extended calibration interval of those algorithms, the follow-up experiment was carried out on eight subjects from the healthy group, with data collected from these subjects while seated at rest the same time of the first day and the next day. Those models were calibrated at the beginning of the first day measurement.

### Data Analysis

The estimate error was calculated as the difference between the reference and the BP estimation, i.e., error = estimated BP – reference BP, and evaluated as the error mean ± standard deviations (SD), as well as mean absolute difference (MAD). They are defined as below:

9 $${\rm{mean}}=\frac{1}{n}\sum _{i=1}^{n}(B{P}_{ES{T}_{i}}-B{P}_{RE{F}_{i}})$$

10 $${\rm{SD}}=\sqrt{\frac{1}{n}\sum _{i=1}^{n}{(B{P}_{ES{T}_{i}}-B{P}_{RE{F}_{i}})}^{2}}$$

11 $${\rm{MAD}}=\frac{1}{n}\sum _{i=1}^{n}|(B{P}_{ES{T}_{i}}-B{P}_{RE{F}_{i}})|.$$

*BP*_*REFi*_ and *BP*_*ESTi*_ represent the *i*th beat BP measured by the reference method and estimated by the cuffless methods, respectively, and *n* is the number of cardiac cycle that used for evaluation. For normotensive and hypertensive group comparison, the student’s *t*-test was used to test the significance between the two groups. One-way analysis of variance (ANOVA) was conducted to test the significance of various methods for BP estimation. The differences between the estimations and the references were tested to be normal distribution. Thus the Tukey’s honestly significant difference was used for comparing group means, and Levene’s test (absolute deviations) was applied for homogeneity of variance test. *p* < 0.05 is taken as statistically significant.
